# Generalized Equilibrium Problem with Mixed Relaxed Monotonicity

**DOI:** 10.1155/2014/807324

**Published:** 2014-07-10

**Authors:** Haider Abbas Rizvi, Adem Kılıçman, Rais Ahmad

**Affiliations:** ^1^Department of Mathematics, Aligarh Muslim University, Aligarh 202002, India; ^2^Department of Mathematics and Institute for Mathematical Research, Universiti Putra Malaysia, 43400 Selangor, Malaysia

## Abstract

We extend the concept of relaxed *α*-monotonicity to mixed relaxed *α*-*β*-monotonicity. The concept of mixed relaxed *α*-*β*-monotonicity is more general than many existing concepts of monotonicities. Finally, we apply this concept and well known KKM-theory to obtain the solution of generalized equilibrium problem.

## 1. Introduction

Generalized monotonicities provide a way of finding parameter moves that yield monotonicity of model solutions and allow studying the monotonicity of functions or subset of variables. In recent past, many researchers have proposed many important generalizations of monotonicity such as pseudomonotonicity, relaxed monotonicity, relaxed *α*-*β*-monotonicity, quasimonotonicity, and semimonotonicity; see [1–3]. Karamardian and Schaible [4] introduced various kinds of monotone mappings which in the case of gradient mappings are related to generalized convex functions. For more details, we refer to [5–7].

Many problems of practical interest in optimization, economics, and engineering involve equilibrium in their description. The techniques involved in the study of equilibrium problems are applicable to a variety of diverse areas and proved to be productive and innovative. Blum and Oettli [8] and Noor and Oettli [9] have shown that the mathematical programming problem can be viewed as special realization of abstract equilibrium problems.

Inspired and motivated by the recent development of equilibrium problems and their solutions methods, in this paper, we extend the concept of relaxed *α*-monotonicity to mixed relaxed *α*-*β*-monotonicity. Finally, this concept is applied with KKM-theory to solve a generalized equilibrium problem. The results of this paper can be viewed as generalization of many known results; see [10–13].

## 2. Preliminaries

Let *K* be a nonempty subset of real Banach space *X*. Let *ϕ* : *K* × *K* → *R* be a real-valued function and let *f* : *K* × *K* → *R* be an equilibrium function; that is, *f*(*x*, *x*) = 0, for all *x* ∈ *K*. We consider the following generalized equilibrium problem: find $\overset{-}{x}\in K$ such that

(1) $$\begin{array}{c} \;f\left(\overset{-}{x},y\right)+\phi \left(\overset{-}{x},y\right)-\phi \left(\overset{-}{x},\overset{-}{x}\right)\geq 0,\;\forall y\in K. \end{array}$$

Problem (1) has been studied by many authors in different settings; see, for instance, [14].

If *ϕ* ≡ 0, then the problem (1) reduces to the classical equilibrium problem, that is, to find $\overset{-}{x}\in K$ such that

(2) $$\begin{array}{c} f\left(\overset{-}{x},y\right)\geq 0,\;\text{with}\;f\left(x,x\right)=0,\;\forall y\in K. \end{array}$$

Problem (2) was introduced and studied by Blum and Oettli [8].

We need the following definition and results in the sequel.

**Definition 1 .**

A real-valued function defined on a convex subset *K* of *X* is said to be hemicontinuous if

(3) $$\begin{array}{c} \underset{t\to 0^{+}}{\lim}f\left(tx+\left(1-t\right)y\right)=f\left(y\right),\;\text{for}\;\text{each}\;x,y\in K. \end{array}$$

**Definition 2 .**

Let *f* : *K* → 2^*X*^ be a multivalued mapping. The *f* is said to be a KKM-mapping if, for any finite subset {*y*_1_, *y*_2_,…, *y*_*n*_} of *K*, co⁡{*y*_1_, *y*_2_,…, *y*_*n*_} ⊂ ⋃_*i*=1_^*n*^ *f*(*y*_*i*_), where co⁡ denotes the convex hull.

**Lemma 3 (see [15]).**

Let *K* be a nonempty subset of a topological vector space *X* and let *f* : *K* → 2^*X*^ be a KKM-mapping. If *f*(*y*) is closed in *X* for all *y* ∈ *K* and compact for at least one *y* ∈ *K*, then ⋂_*y*∈*K*_ *f*(*y*) ≠ *ϕ*.

**Definition 4 .**

Let *X* be a Banach space. A mapping *f* : *X* → *R* is said to be lower semicontinuous at *x*_0_ ∈ *X*, if

(4) $$\begin{array}{c} f\left(x_{0}\right)\leq \underset{n}{\lim}\inf f\left(x_{n}\right), \end{array}$$

for any sequence {*x*_*n*_} of *X* such that *x*_*n*_ → *x*_0_.

**Definition 5 .**

Let *X* be a Banach space. A mapping *f* : *X* → *R* is said to be weakly upper semicontinuous at *x*_0_ ∈ *X*, if

(5) $$\begin{array}{c} f\left(x_{0}\right)\geq \underset{x}{\lim}\sup f\left(x_{n}\right), \end{array}$$

for any sequence {*x*_*n*_} of *X* such that *x*_*n*_ → *x*_0_.

Now, we extend the definition of relaxed *α*-monotonicity [11] to mixed relaxed *α*-*β*-monotonicity.

**Definition 6 .**

A mapping *f* : *K* × *K* → *R* is said to be mixed relaxed *α*-*β*-monotone, if there exist mappings *α* : *K* → *R* with *α*(*tx*) = *t* ^*p*^ *α*(*x*), for all *t* > 0 and *β* : *K* × *K* → *R*, such that

(6) $$\begin{array}{c} f\left(x,y\right)+f\left(y,x\right)\leq \alpha \left(y-x\right)+\beta \left(x,y\right),\;\forall x,y\in K, \end{array}$$

where

(7) $$\begin{array}{c} \underset{t\to 0}{\lim}\left[\frac{t^{p}\alpha \left(y-x\right)}{t}+\frac{\beta \left(x,ty+\left(1-t\right)x\right)}{t}\right]=0, \end{array}$$

and *p* > 1 is a constant.

If *β* = 0, then Definition 6 reduces to the definition of generalized relaxed *α*-monotone; that is,

(8) $$\begin{array}{c} f\left(x,y\right)+f\left(y,x\right)\leq \alpha \left(y-x\right),\;\forall x,y\in K, \end{array}$$

where

(9) $$\begin{array}{c} \underset{t\to 0}{\lim}\left[\frac{t^{p}\alpha \left(y-x\right)}{t}\right]=0,\;p>1\;\text{is}\;\text{a}\;\text{constant}. \end{array}$$

If *α* = 0, then Definition 6 reduces to the definition of generalized relaxed *β*-monotone; that is,

(10) $$\begin{array}{c} f\left(x,y\right)+f\left(y,x\right)\leq \beta \left(x,y\right),\;\forall x,y\in K, \end{array}$$

where

(11) $$\begin{array}{c} \underset{t\to 0}{\lim}\frac{\beta \left(x,ty+\left(1-t\right)x\right)}{t}=0. \end{array}$$

If both *α* = 0 = *β*, then Definition 6 coincides with the definition of monotonicity; that is,

(12) $$\begin{array}{c} f\left(x,y\right)+f\left(y,x\right)\leq 0,\;\forall x,y\in K. \end{array}$$

**Definition 7 .**

A mapping *ϕ* : *K* × *K* → *R* ∪ {±0} is said to be *o*-diagonally convex if, for any finite subset {*x*_1_, *x*_2_,…, *x*_*n*_} of *K* and *λ*_*i*_ ≥ 0  (*i* = 1,2,…, *n*) with ∑_*i*=1_^*n*^ *λ*_*i*_ = 1 and $\overset{-}{x}=\sum_{i=1}^{n}\lambda_{i}x_{i}$, one has

(13) $$\begin{array}{c} \overset{n}{\underset{i=1}{\sum }}\lambda_{i}\phi \left(\overset{-}{x},x_{i}\right)\geq 0. \end{array}$$

## 3. Existence of Solution for Generalized Equilibrium Problem

We establish this section with the discussion of existence of solution for generalized equilibrium problem by using mixed relaxed *α*-*β*-monotonicity.

**Theorem 8 .**

Suppose *f* : *K* × *K* → *R* is mixed relaxed *α*-*β*-monotone, hemicontinuous in the first argument and convex in the second argument with *f*(*x*, *x*) = 0, for all *x* ∈ *K*. Let *ϕ* : *K* × *K* → *R* be convex in the second argument. Then, generalized equilibrium problem (1) is equivalent to the following problem.

Find $\overset{-}{x}\in K$ such that

(14) f(y,x−)+ϕ(x−,x−)−ϕ(x−,y)≤α(y−x−)+β(x−,y), ∀y∈K,

where *α*(*tx*) = *t* ^*p*^ *α*(*x*) and *p* > 1 is a constant.

**Proof**

Suppose that the generalized equilibrium problem (1) admits a solution; that is, there exists $\overset{-}{x}\in K$ such that

(15) $$\begin{array}{c} f\left(\overset{-}{x},y\right)+\phi \left(\overset{-}{x},y\right)-\phi \left(\overset{-}{x},\overset{-}{x}\right)\geq 0,\;\forall y\in K. \end{array}$$

Since *f* is mixed relaxed *α*-*β*-monotone, we have

(16) $$\begin{array}{c} f\left(\overset{-}{x},y\right)+f\left(y,\overset{-}{x}\right)\leq \alpha \left(y-\overset{-}{x}\right)+\beta \left(\overset{-}{x},y\right),\;\forall y\in K, \end{array}$$

(17) $$\begin{array}{c} f\left(y,\overset{-}{x}\right)\leq \alpha \left(y-\overset{-}{x}\right)+\beta \left(\overset{-}{x},y\right)-f\left(\overset{-}{x},y\right),\;\forall y\in K. \end{array}$$

Adding $\phi (\overset{-}{x},\overset{-}{x})-\phi (\overset{-}{x},x)$ on both sides of (17), we have

(18) f(y,x−)+ϕ(x−,x−)−ϕ(x−,y)  ≤α(y−x−)+β(x−,y)−[f(x−,y)+ϕ(x−,y)−ϕ(x−,x)]  ≤α(y−x−)+β(x−,y), ∀y∈K.

Hence, $\overset{-}{x}\in K$ is a solution of problem (14).

Conversely, suppose that $\overset{-}{x}\in K$ is a solution of problem (14); that is,

(19) f(y,x−)+ϕ(x−,x−)−ϕ(x−,y)≤α(y−x−)+β(x−,y), ∀y∈K.

Let $x_{t}=ty+(1-t)\overset{-}{x}$, *t* ∈ [0,1], and *y* ∈ *K*; then clearly *x*_*t*_ ∈ *K* as *K* is convex. Thus from (17), we have

(20) $$\begin{array}{c} f\left(x_{t},\overset{-}{x}\right)+\phi \left(\overset{-}{x},\overset{-}{x}\right)-\phi \left(\overset{-}{x},x_{t}\right)\leq \alpha \left(x_{t}-\overset{-}{x}\right)+\beta \left(\overset{-}{x},x_{t}\right). \end{array}$$

Since *f* is convex in the second argument, we have

(21) $$\begin{array}{c} 0=f\left(x_{t},x_{t}\right)\leq tf\left(x_{t},y\right)+\left(1-t\right)f\left(x_{t},\overset{-}{x}\right) \end{array}$$

which implies that

(22) $$\begin{array}{c} t\left[f\left(x_{t},\overset{-}{x}\right)-f\left(x_{t},y\right)\right]\leq f\left(x_{t},\overset{-}{x}\right). \end{array}$$

Also as *ϕ* is convex in the second argument, we have

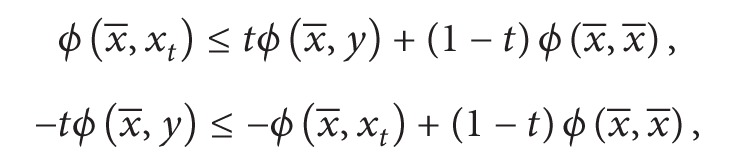
(23)

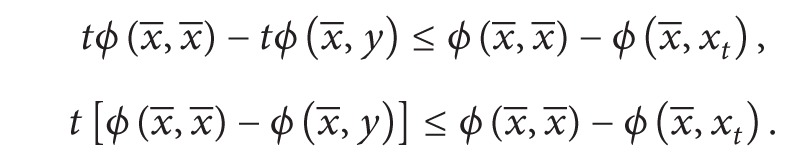
(24)

Adding (22) and (24), we have

(25) t[f(xt,x−)−f(xt,y)+ϕ(x−,x−)−ϕ(x−,y)]  ≤f(xt,x−)+ϕ(x−,x−)−ϕ(x−,xt)  ≤α(xt−x−)+β(x−,xt).

It follows that

(26) f(xt,x−)−f(xt,y)+ϕ(x−,x−)−ϕ(x−,y)  ≤α(xt−x−)t+β(x−,xt)t  ≤tpα(y−x−)t+β(x−,xt)t, p>1.

Since *f* is hemicontinuous in the first argument, taking *t* → 0, we have

(27) $$\begin{array}{c} f\left(\overset{-}{x},\overset{-}{x}\right)-f\left(\overset{-}{x},y\right)+\phi \left(\overset{-}{x},\overset{-}{x}\right)-\phi \left(\overset{-}{x},y\right)\leq 0; \end{array}$$

that is, we have

(28) $$\begin{array}{c} f\left(\overset{-}{x},y\right)+\phi \left(\overset{-}{x},y\right)-\phi \left(\overset{-}{x},\overset{-}{x}\right)\geq 0,\;\forall y\in K. \end{array}$$

Hence $\overset{-}{x}\in K$ is a solution of generalized equilibrium problem (1).

**Theorem 9 .**

Let *K* be a nonempty bounded closed convex subset of a real Banach space *X*. Let *f* : *K* × *K* → *R* be a mixed relaxed *α*-*β*-monotone, hemicontinuous in the first argument, convex in the second argument with *f*(*x*, *x*) = 0, *o*-diagonally convex, and lower semicontinuous. Let *ϕ* : *K* × *K* → *R* be convex in the second argument, *o*-diagonally convex, and lower semicontinuous; *α* : *K* → *R* is weakly upper semicontinuous and *β* : *K* × *K* → *R* is weakly upper semicontinuous in the second argument. Then the mixed equilibrium problem (1) admits a solution.

**Proof**

Consider a multivalued mapping *F* : *K* → 2^*X*^ such that

(29) F(y)={x−∈K:f(x−,y)+ϕ(x−,y)−ϕ(x−,x−)≥0},∀y∈K.

We show that ⋂_*y*∈*K*_ *F*(*y*) = *ϕ*; that is, $\overset{-}{x}\in K$ is a solution of generalized equilibrium problem (1).

Our claim is that *F* is a KKM-mapping. Suppose to contrary that is *F* is not a KKM-mapping; then there exists a finite subset {*x*_1_, *x*_2_,…, *x*_*n*_} of *K* and *λ*_*i*_ ≥ 0  (*i* = 1,2,…*n*) with ∑_*i*=1_^*n*^ *λ*_*i*_ = 1 such that

(30) $$\begin{array}{c} x_{0}=\overset{n}{\underset{i=1}{\sum }}\lambda_{i}x_{i}\notin \overset{n}{\underset{i=1}{⋃}}F\left(y_{i}\right). \end{array}$$

It follows that

(31) $$\begin{array}{c} f\left(x_{0},x_{i}\right)+\phi \left(x_{0},x_{i}\right)-\phi \left(x_{0},x_{0}\right)<0,\;\text{for}\;i=1,2,\ldots ,n. \end{array}$$

Also we have

(32) ∑i=1nλi[f(x0,xi)+ϕ(x0,xi)−ϕ(x0,x0)]<0,for  i=1,2,…,n,

which contradicts the *o*-diagonal convexity of *f* and *ϕ*. Hence *F* is a KKM-mapping.

Now consider another multivalued mapping *G* : *K* → 2^*X*^ such that

(33) G(y)={x−∈K:f(y,x−)+ϕ(x−,x−)−ϕ(x−,y)≤α(y−x−)+β(x−,y)}, ∀y∈K.

We will show that *F*(*y*) ⊂ *G*(*y*), ∀*y* ∈ *K*. For any given *y* ∈ *K*, let $\overset{-}{x}\in F(y)$; then

(34) $$\begin{array}{c} f\left(\overset{-}{x},y\right)+\phi \left(\overset{-}{x},y\right)-\phi \left(\overset{-}{x},\overset{-}{x}\right)\geq 0. \end{array}$$

It follows from the mixed relaxed *α*-*β*-monotonicity of *f* that

(35) f(y,x−)+ϕ(x−,x−)−ϕ(x−,y)  ≤α(y−x−)+β(x−,y)−[f(x−,y)+ϕ(x−,y)−ϕ(x−,x−)]  ≤α(y−x−)+β(x−,y);

that is, $\overset{-}{x}\in G(y)$. Thus *F*(*y*) ⊂ *G*(*y*) and consequently *G* is also KKM-mapping.

Since *f* and *ϕ* both are convex in the second argument and lower semicontinuous, thus they both are weakly lower semicontinuous. From weakly upper semicontinuity of *α*, weakly upper semicontinuity of *β* in the second argument, and the construction of *G*, it is accessible to see that *G*(*y*) is weakly closed for all *y* ∈ *K*. Since *K* is closed, bounded, and convex, it is weakly compact and consequently *G*(*y*) is weakly compact in *K* for all *y* ∈ *K*. Therefore, from Lemma 3 and Theorem 8, we have

(36) $$\begin{array}{c} \underset{y\in K}{⋂}F\left(y\right)=\underset{y\in K}{⋂}G\left(y\right)\neq \phi ; \end{array}$$

that is, there exists $\overset{-}{x}\in K$ such that

(37) $$\begin{array}{c} f\left(\overset{-}{x},y\right)+\phi \left(\overset{-}{x},y\right)-\phi \left(\overset{-}{x},\overset{-}{x}\right)\geq 0,\;\forall y\in K. \end{array}$$

Thus, the generalized equilibrium problem (1) admits a solution.
